# Current Government Actions and Potential Policy Options for Reducing Obesity in Queensland Schools

**DOI:** 10.3390/children5020018

**Published:** 2018-01-29

**Authors:** Naser A. Alsharairi

**Affiliations:** Understanding Chronic Conditions, Heart, Mind & Body Research Group, Menzies Health Institute Queensland, Griffith University, Gold Coast Campus, Southport, QLD 4222, Australia; naser.alsharairi@gmail.com

**Keywords:** policy options, obesity, children, Queensland schools

## Abstract

School nutrition policies provide promising avenues towards the improvement of children’s eating habits and the prevention of obesity. Childhood obesity rates and related chronic diseases are increasing in Queensland, in part as a result of unhealthy eating habits and lack of physical activity. There is a very high investment by the Queensland government in maintaining healthy weight and promoting nutrition and physical activity among schoolchildren through delivering a range of initiatives across the state. However, there is a lack of evidence concerning the effectiveness of nutrition/physical education and parental involvement programs addressing obesity delivered in Queensland schools. This paper can be used to guide government and policy-makers regarding the most effective policy options that will promote healthy eating and physical activity among Queensland schoolchildren. The aim of this paper is to: (i) summarize current evidence on Queensland government responses to obesity; and (ii) discuss potential policy options that could support healthy eating and regular physical activity, and examine the evidence base for each option and suggest new areas for future research.

## 1. Introduction

The prevalence of childhood obesity worldwide continues to increase, with substantial implications for the associated risk of chronic cardiometabolic disease such as diabetes [1]. Obesity is a major public health issue in Australia, and the healthcare costs for children with obesity are high [2]. Childhood obesity rates in Australia have increased contemporaneously with that of adults, with nearly a quarter of children now considered to be overweight or obese [3]. The prevalence of obesity has reached an alarming level among schoolchildren in Australia [4,5,6]. A cross-sectional study carried out on a sample of 31,424 schoolchildren in five Australian states indicated that 24.6% of children aged 4.5–15 years were overweight or obese [7]. The study also showed that children were classified into six body mass index (BMI) categories according to the International Obesity Task Force age- and sex-specific criteria cutoff point: severe thinness, moderate thinness, mild thinness and normal weight (BMI 16–18.5 kg/m^2^), overweight (equivalent to BMI 25 kg/m^2^) and obese (equivalent to BMI 30 kg/m^2^) [8,9].

Childhood obesity is a critical health issue in Queensland, particularly in disadvantaged and remote areas [10]. Townsville, West Moreton, Darling Downs and Mackay reported the highest rates of obesity and overweight across Queensland [11]. According to the Australian Health Survey 2011–2012, the prevalence of overweight and obesity in Queensland children aged 5–17 were 18.2% and 9.3% respectively [12]. The most recent survey (2014–2015) indicates that the prevalence of overweight and obesity in Queensland children was about 19% and 7%, respectively, equating to about 217,000 children [11].

Foods with a high energy density are key contributors to obesity in Australian schoolchildren [13]. In Queensland, 61% of children aged 5–17 years consumed ‘discretionary’ foods (i.e., savoury biscuits, salty snacks, confectionary) and half of children (51%) aged 2–18 years consumed sugar-sweetened drinks daily [11]. A high proportion of children (70%) did eat the recommended daily amount of fruit. Conversely, less than 10% of children (3.7%) ate the recommended daily amount of vegetables [11]. Limited evidence indicates that schoolchildren aged 11–12 years in Queensland skipped breakfast [14]. Lack of physical activity in schools also plays a role in childhood obesity [15,16]. A Healthy Kids Queensland Survey 2006 reported that under half of children (*n* = 2640) aged 6–10 years did meet the guidelines for physical activity of 60 min or more per day [17]. The prevalence of being physically inactive every day in Queensland was 41% and 73% among children aged 5–7 years and 16–17 years, respectively [11].

Policy refers to practices or rules of actions that apply to many sectors to achieve desirable goals [18]. State governments are responsible for developing and evaluating nutrition education policy and goal-setting for achieving positive health outcomes for children [19]. Increasing obesity rates among schoolchildren highlights the need for policies that support healthy eating and regular physical activity in schools [20]. Policy plays a major role in beliefs and values that shape the personal and environmental determinants of behaviours, and through these determinants can induce behaviour change [21]. Policy can influence the environmental determinants by delineating a variety of instruments to allay adverse circumstances and to provide opportunities for health and to reduce obesity [21,22]. For example, taxes on high-calorie food items or beverages and rules for bike paths to maximize their environmental impact may reduce obesity [23]. In the UK, the Department of Health and Social Care has developed action plans to reduce obesity among schoolchildren. These include introducing a new levy on soft drinks that contain added sugar, establishing high-quality sport and physical activity programs, creating a new healthy rating scheme for primary schools and implementing the School Food Plan to improve school food and schoolchildren’s diets [24]. Policy can also influence the personal determinants as well as education by recommending beneficial health behaviours to replace unhealthy behaviours [21]. For example, the New South Wales (NSW) government has established a policy “fresh tastes healthy canteens strategy” aimed to control canteen menus and to reduce obesity in public schools. The NSW government action plan is to provide healthier food items in accordance with the ideologies of the Australian Dietary Guidelines [25]. The World Health Organisation has proposed beneficial policy options in the school environment. These policies contain statements to support healthy eating and regular physical activity in schools, ban food advertising in schools, an inclusive education for children on healthy food choices, increase the number of physical education classes and the implementation of the national/regional food-based dietary guidelines for schoolchildren [26]. In the U.S., the Centers for Disease Control and Prevention (CDC) has developed strategies to promote healthy eating and physical activity in schools. These include providing access to healthy foods and safe spaces for active play, encouraging children to participate in physical activity outside of physical education class and providing children with health education classes that support healthy eating [27].

This paper illustrates the argument that obesity prevention policy in Queensland schools is a challenge [28]. The Queensland government has made significant investments in promoting healthy foods and drinks to children in schools [29]. However, there is still much to do, as children are still eating too many snack foods, and are physically inactive. The current evidence of policy is limited to the role of Queensland government in reducing access to unhealthy foods in school tuckshops and increasing children’s physical activity levels [28,30,31]. There are, however, other policies requiring further investigation. These may include the development of policy options focused on nutrition/physical education and/or parental involvement in school nutrition programs that could potentially result in reducing obesity among schoolchildren in Queensland. The empirical evidence on personal and environmental determinants of children’s eating and physical activity behaviour is relatively sparse in relation to which aspects of the food and physical activity environment are most influential, and how environmental factors interact with individual factors. Further, there is still inadequate knowledge of the most effective policies for improving healthy eating and physical activity in schools [32,33]. Interventions incorporating environmental change components (i.e., parental involvement in school-based child nutrition programs) and individual behaviour change components can be effective in promoting healthy behaviours among schoolchildren. School-based nutrition education programs were found to be effective in promoting attitude and self-efficacy and increasing nutrition knowledge and preferences for healthy foods and physical activity among schoolchildren [34,35,36]. Research into parental involvement suggests that the intervention programs in which parents were encouraged to participate in a variety of meaningful multi-component activities such as the classroom curriculum have the greatest impact upon improving healthy eating habits and physical activity in children [37,38,39]. This paper aims to: (i) summarize current evidence on Queensland government responses to obesity; and (ii) discuss potential policy options that could reduce childhood obesity in Queensland schools.

## 2. Queensland Government Responses to Obesity

At the Queensland Obesity Summit in 2006, the Premier allocated $21 million to be used in grant funding, partnership and other recourses to promote healthy eating habits and increase physical activity. Following the summit, the Premier has established the Obesity Taskforce and Eat Well, Be Active as positive initiatives towards preventing obesity in schools [17]. Eat Well, Be Active-Healthy Kids for Life Action Plan 2005–2008 was the first initiative to address the problem of obesity and overweight in Queensland schools. It aimed to promote healthy diets and physical activity to all children across Queensland [29]. Many initiatives, relating to unhealthy eating and lack of physical activity come under the flagship policy of the Eat Well policy such as the Active-Ate Program, Eat Well Queensland, the Physical Activity and Nutrition Out of School Hours Care Program (PANOSH) program, Smart Choices and Smart Moves. The Active-Ate Program conducted by Queensland Health aimed to increase the availability of healthy foods at school canteens, including the school breakfast program, and by so doing, reduce obesity and diabetes among children. The program included school resources and a classroom component for teachers to achieve the goal of the program [40]. Eat Well Queensland 2002–2012 was developed to assist with weight loss and promote healthy diets and physical activity among Indigenous, disadvantaged people and schoolchildren. The goal of the strategy was to disseminate messages about healthy diets and physical activity to the public through social media and development of an advocacy plan. The priority area of activity was weight reduction by increasing the availability of healthy foods and reducing high-energy-dense foods in school canteens and increasing children’s physical activity levels [41]. Queensland Health also implemented the PANOSH program, which aimed to have healthy foods available and affordable out of the school hours and to enable children to stay active after school and during vacation time [17]. The program provided healthy foods which align with the Australian Dietary Guidelines and the Australian Guide to Health Eating. The program also encouraged children to participate for at least 30 min every day in a range of activities such as running, jumping, pushing, climbing and dancing games [42].

In 2007, Smart Choices 2004–2007—Healthy Food and Drink Supply Strategy for Queensland Schools was implemented as a part of the Queensland Government agenda to reduce the prevalence of obesity and overweight in schoolchildren. It asserted that food and drinks supplied in the school environment (i.e., the tuckshop, vending machines, sports days, school events, school excursions, classroom rewards, fundraising, curriculum activities) should align with the National Health and Medical Research Council’s Dietary Guidelines for Children and the Australian Guide to Healthy Eating. Under this scheme, food and drinks are classified as GREEN (Healthy foods, good source of many nutrients), AMBER (select carefully, have limited nutritional value) or RED (select occasionally, part of poor and inadequate diets). The implementation of Smart Choices in Queensland Schools was supported by Queensland Health; school tuckshops, Nutrition Australia and the Department of Education and Training. The evaluation of the Smart Choices program indicated that a self-report was an appropriate tool of implementation. The implementation tended to be slightly higher in urban and secondary schools than in rural and primary schools [28,30]. *Smart Moves* has been implemented as a companion initiative to Smart Choices aiming to increase physical activity levels among primary and secondary school children. Key recommendations of the *Smart Moves* programme include: (1) primary and secondary schools are required to allocate 30 min per day and 2 h per week to physical activity as part of the school curriculum respectively; (2) creating a partnership between schools and sporting organizations to increase children’s participation in physical activity; (3) providing professional development for principals and physical education teachers to increase the delivery of physical activity to children; and (4) improving access to sport and recreation facilities can provide benefit to both the school and community [31,43].

## 3. Policy Options for Reducing Obesity in Queensland Schools

The present study identifies some of the policy options that could be effective in reducing obesity among schoolchildren in Queensland. These options are: nutrition education, physical education and parental involvement in nutrition and physical activities. Importantly, this paper did not assess the policy options of its content and capacity for implementation or set evaluation indicators, so there is no precise measure to indicate that these options will achieve the desired outcomes. Each option is to be described in a separate section.

### 3.1. Nutrition Education

Nutrition education may include nutrition workshops and interactive nutrition lessons. In Australia, evidence found that implementing school-based nutrition education programs in NSW was effective in promoting healthy eating among children [44,45]. Children engaged in nutrition activities in their school lessons demonstrated positive changes in knowledge, self-efficacy and attitude towards eating healthy foods, suggesting that these strategies have positive implications for the dietary behaviours of children in schools [34]. A 12-week program included participation in interactive activities and a classroom curriculum that encouraged increased fruit and vegetable (FV) intake in 14 urban low-income schools in Louisiana, USA, found that children acquired significant knowledge of healthy nutrition practices and became confident that they would choose to eat fruit and vegetables instead of dessert [46]. A randomized controlled intervention study comprising 10 classroom-based nutrition lessons (including, for example, a fruit and vegetable serving size poster, a fruit and vegetable song audio tape, cook books, and teacher lesson plan) run over an 8-week period in 31 low-income public schools in California, USA, found that the intervention had a successful outcome, showing improvements in children’s self-efficacy, knowledge, and outcome expectancies relating to fruit and vegetable intake [47]. An intervention study was designed to increase core food consumption (i.e., fruit and vegetables, dairy foods, and meat) and to reduce non-core food consumption (i.e., baked snacks, cakes, cookies) among adolescent girls in 12 low-income secondary schools in NSW, Australia. The intervention activities included interactive seminars, nutrition handbooks, and nutrition workshops. Results showed that girls had more self-efficacy to increase core food consumption and decrease non-core food consumption [48].

School gardens have potential as a promising strategy for reducing childhood obesity, particularly in relation to increasing access to fruits and vegetables and improving children’s physical activity [49]. School gardens may increase physical activity by increasing in sedentary activities outdoors and engaging children in hands-on gardening activities [50]. School gardens are an integral component of nutrition education policy that has resulted in not only increased fruit and vegetable intake, but also increased nutrition knowledge and preference for fruit and vegetables in children [51]. There is some evidence to support the educational effectiveness of school gardens in Australia. Previous evidence in Queensland has shown the effectiveness of school gardens in improving children’s attitudes towards eating fruit and vegetables, but in the long term may require additional resources for the establishment of gardens and for identifying factors affecting the implementation of school gardens [52]. Other evidence in NSW showed that school gardens are effective in increasing nutrition knowledge and preferences for fruit and vegetables, but the program was short in duration, resulting in small dietary behaviour changes among children [44,53]. School gardens have been shown positively to change children’s preferences, self-efficacy and attitude for fruit and vegetable consumption [54]. A study examined the impact of a school garden intervention on preferences for FV consumption among Aboriginal children in grades 1–6 in Alberta, Canada. Children planted fruit and vegetables in a classroom container garden and ate what they grew. The taste preferences for fruit and vegetables were calculated using a Likert scale at baseline and 7 months later. The study found that children after the 7-month intervention program had a significantly higher preference score for fruit and vegetables than at the baseline [55]. A 12-week garden-based nutrition intervention program was designed to increase fruit and vegetable intake among 4th to 6th grade children in Rochester, Minnesota, USA. The intervention activities included a newsletter sent home, preparation of fruit and vegetable snacks and fruit and vegetable taste tests. The process of intervention included collecting data from four open-ended questions asking children to explain what they liked and disliked about the intervention. Results showed that that the program was successful in significantly influencing children’s vegetable preferences [56]. A school-based gardening intervention was designed to increase vegetable intake in 6th grade children in San Francisco, USA. Schools were assigned to 1 comparison and 2 intervention groups. Willingness to eat vegetables, along with attitudes towards and preferences for vegetables were the three learning processes. The assessment used self-administered surveys (the Garden Vegetable Frequency Questionnaire) with children and a taste test to measure consumption and preferences for vegetables. Such intervention has been successful in improving children’s preferences for, willingness to, and attitudes towards eating vegetables compared with children in the comparison school [57]. A multi-component garden school-based intervention was conducted among 6th and 7th grade children in 5 ethnic, low-income schools in Austin, Texas, USA. Schools were assigned to 4 interventions and 1 control group. The intervention comprised six components (farm to school, taste testing, in-class lessons, field trips to farms, after-school gardening program, and farmer’s visit to school). Learning processes included knowledge, self-efficacy, motivation and preferences for fruit and vegetable, and preferences for unhealthy foods. The main results showed that children who were exposed to two or more components ate more fruit and vegetables, had more knowledge and self-efficacy to eat fruit and vegetables and had lower preferences for unhealthy foods [58].

### 3.2. Physical Education

Physical activity is essential for improving the fitness of children and reducing the risk of obesity, diabetes and cardiovascular diseases [59]. The recommendation is for Australian children aged 5–17 years to do moderate or vigorous physical activity for 60 min per day [60]. In general, Australian children aged 5–17 years participated in organised sports more than active transportation and school-based physical activity. Given that 80% of Australian children are estimated to be physically inactive, participation in other forms of physical activities are needed to ensure that children are physically active [61].

Physical activity policies that require curriculum, infrastructure resources, and time allocations for physical education in schools, and that make physical activity the easiest choice for children, are important factors in making school environments healthier spaces for children. However, policies alone are not currently at a sufficient level to address the physical inactivity of children at school. Physical activity policies should also focus on individual factors that could potentially result in more successful outcomes. For example, teacher involvement with activities involving children in physical education classes may have implications for promoting physical activity behaviour in children and sustaining the improvement of intervention programs within schools [62]. A randomized controlled trial aimed to explore the potential mediators of physical activity in 5th and 6th grade children in the Hunter region (NSW, Australia) at baseline and at 6-month follow-up. The intervention was successful in significantly improving physical activity by the end of the program. Teacher social support was found to have a mediating effect on physical activity. The study suggests that social support for physical activity provided by classroom teachers should be considered as a potential strategy to improve children’s physical activity [63]. The goal of a quasi-experimental intervention study was to evaluate a 1-year teaching intervention which supported teachers in increasing children’s moderate-to-vigorous physical activity (MVPA) in the West Midlands, UK. Children were assigned to one intervention school and one control school. The intervention included children’s activity levels (standing, walking, sitting, lying, dance, swimming), lesson context (fitness, games, management, knowledge) and teacher promotion of physical activity (in-class promotion, out-class promotion or no promotion) at baseline and post-intervention in each school. Post-intervention, children in the intervention school engaged in significantly more MVPA during physical activity lessons than children in the control school. Teachers' promotion of physical activity was found to be significantly greater in the intervention school than in the control school [64]. Research has demonstrated that effective health educational interventions improved attitude and self-efficacy, increased knowledge or belief and enjoyment or preference for physical activity among schoolchildren [35,36,65,66].

### 3.3. Parental Involvement

Parental involvement in daily lifestyle activities has been found to improve children’s dietary intake [67]. Research has shown that parental involvement in activities (i.e., weekly newsletters, text messages, phone calls, food preparation, meetings) are critical features of successful school nutrition programs [54,68,69,70]. The school nutrition programs have been proven effective in NSW, Australia, but require school orientation programs with parents to promote children’s healthy dietary habits in schools [45]. Parental involvement in a multi-component school-based intervention was found to be effective in promoting health eating habits and physical activity in children [37,38,39,71]. Parents involved in school nutrition programs can serve as positive role models, and thus improve children’s healthy eating and physical activity behaviours. Parents who participated in healthy lifestyle activities (i.e., engaging in physical activity, reducing screen time) may themselves have developed some of the healthier food behaviours, and in turn children may have mirrored their parents’ lifestyle activities [72,73,74].

## 4. Conclusions and Recommendations for Future Research

This paper can be used to guide future research directions and influence policy decisions to reduce obesity in Queensland schools. Policy change can address the social determinants of health. Social determinants of health are social and economic conditions that influence health-related behaviours [75]. As such, policy aims attention at changes from behaviour itself to the processes and forces that create patterns of behaviour [76].

The prevalence of obesity and overweight among schoolchildren in Queensland is still too high. The most effective policy for reducing childhood obesity in Queensland schools is arguably to limit access to unhealthy foods and drinks, and replace them with healthier food choices, and increase children’s physical activity levels. The Queensland government has been responding to the obesity problem. It is important that attention is now directed towards implementing more policy responses to address the obesity problem in Queensland schools. A more strategic response needs to make use of policy engagement strategies by members of the advocacy coalition, or by informing stakeholders (e.g., coordinators, teachers, volunteers) about the benefits of promoting healthy diets and increasing physical activity [77]. In NSW and Western Australia, the engagement of parents and teachers as stakeholders in school-based programs is important for promoting children’s healthy eating and physical activity [78,79]. The goal of an Australian study was to identify factors of stakeholders’ perceptions (school principals, parents) of the impact of a ‘Healthy Food and Drink Policy’ on schoolchildren’s diet in Western Australia. The factors that facilitate implementation include the provision of information skills prior to implementation and the provision of training and assistance for canteen managers [80]. In the Netherlands, stakeholders including parents, teachers and principals take part in supporting the implementation of school food policy and fostering children’s healthy eating habits [81]. In the Canadian context, stakeholders including parents, non-governmental organizations, school personnel and private sectors are key parts of implementing and developing school policies that promote healthy eating and physical activity. Potential roles for the parents include involving in program elements and participating in extracurricular activities at the school. School personnel such as teachers and school administrators are responsible for providing adequate resources and delivering the nutrition education component of a school health program. Non-governmental organizations have a crucial role for implementing school food guidelines, supporting the development of school food gardens and school intramural physical activity. Private sectors play a particularly critical role by advocating for policy changes to improve diets, supporting the development of school physical activity programs and communicating healthy living messages to children [82]. Effective policy needs to be focused on developing an integrated school action plan that includes assessment, implementation and evaluation to reduce obesity among schoolchildren. Such policy may provide useful insights for government to develop more effective policies to improve healthy eating habits and increase physical activity. Effectiveness, efficiency, equity and feasibility are criteria for choosing policy options [18]. Government departments and agencies in both the USA and UK have taken range of policy actions (assessment, implementation, monitoring and evaluation) to improve the dietary and physical activity behaviours of schoolchildren [27,83]. In Australia, nutrition and physical activity policies in all states and territories do not meet expert recommendations and fail to address detailed monitoring and evaluation [84]. In Sweden, an innovative web-based system called SkolmatSverige (School Food Sweden) was developed by semi-governmental bodies and organizations to allow primary schools to undertake their own evaluation and monitoring and thereby improve the quality of school food [85]. Policy-makers will be needed to support policy development, monitor and evaluate policy influence and advocacy, formulate more strategic actions to promote public health and to employ their theories and concepts in policy-making practices [21]. In New Zealand, policy-makers supported the role of schools in promoting nutrition and suggested that state policies should be implemented in schools to develop effective nutrition programs [86]. Policy-makers are often needed to close the evidence gap by providing a clear link between theory and practice. Theories need to be clearly formulated to understand the relationship between environmental and personal-cognitive determinants, and policy [21]. Policy-makers are also needed to decide which approach to policy implementation is most effective to reduce childhood obesity in Queensland schools. Policy advocates of midstream approaches (behavioural approaches) to obesity prevention are needed to address policy interventions targeted at behaviour change among children, aiming to promote healthy eating and physical activity behaviours by using policy tools such as nutrition education strategies [87].

The Queensland Government should provide supportive policy options to reduce obesity in Queensland schools. These may include the development of policy options focused on nutrition/physical education and parental involvement in nutrition and physical activities. Policy responses to obesity in Queensland schools may include developing engagement strategies by increasing communication between stakeholders and policy-makers to achieve the goals of: (1) developing a nutrition and physical education program to improve children’s self-efficacy, knowledge, awareness, outcome expectations, attitudes and preferences for eating healthy foods and doing physical activity; and (2) implementing parent engagement strategies to promote healthy eating and physical activity behaviours among children by keeping parents involved in nutrition and physical education activities. The development of policy recommendations is needed from government regarding nutrition/physical activity education in several broad areas: (1) the use of posters in the classroom to teach children about healthy food and to encourage them to be physically active; (2) the provision of information skills such as recipe books or picture cards that increase children’s awareness and knowledge of healthy eating and physical activity; (3) giving children the opportunity to practice important skills such as food preparation and the opportunity to taste different kinds of healthy foods; (4) the involvement of teachers in the development of policy, and teaching them about theories of behaviour change to assist in maximizing children’s knowledge of activities during class time; (5) the provision of training to teachers on topics such as healthy eating, food safety and good physical activity habits; (6) engaging children and teachers in hands-on gardening activities such as planting, weeding and harvesting foods; and (7) the provision of a broad variety of physical activities such as active play, active transportation, after-school activities, non-competitive recreation and sports to help children achieve a healthy body weight. In addition, the nature of the government actions to implement a parental involvement policy in Queensland schools may take several forms; i.e., (1) to engage parents in nutrition and physical activities to promote healthy eating and physical activity in their children (e.g., the preparation of healthy meals, parents eating with children, walking or running to school with children, taking a walk after meals with children, cycling with children); (2) providing parents with information about healthy eating and physical activity through weekly newsletters; (3) providing parents with information on the types of foods and frequency of their children’s purchases from the canteen along with nutrition messages about encouraging healthy eating habits; and (4) encouraging parents to be part of decision-making related to healthy school nutrition and the physical activity program.
